# Pulmonary transcriptome analysis in the surgically induced rabbit model of diaphragmatic hernia treated with fetal tracheal occlusion

**DOI:** 10.1242/dmm.021626

**Published:** 2016-02-01

**Authors:** Alexander C. Engels, Paul D. Brady, Molka Kammoun, Julio Finalet Ferreiro, Philip DeKoninck, Masayuki Endo, Jaan Toelen, Joris R. Vermeesch, Jan Deprest

**Affiliations:** 1 Department of Development and Regeneration, Organ System Cluster, Faculty of Medicine, KU Leuven, 3000 Leuven, Belgium; 2 Clinical Department of Obstetrics and Gynaecology, Division Woman and Child, University Hospitals KU Leuven, 3000 Leuven, Belgium; 3 Department of Human Genetics, Centre for Human Genetics, University Hospitals KU Leuven, 3000 Leuven, Belgium; 4 Clinical Department of Pediatrics, Division Woman and Child, University Hospitals KU Leuven, 3000 Leuven, Belgium

**Keywords:** RNA-seq, Congenital diaphragmatic hernia, CDH, Pulmonary hypoplasia, Tracheal occlusion, Fetoscopy

## Abstract

Congenital diaphragmatic hernia (CDH) is a malformation leading to pulmonary hypoplasia, which can be treated *in utero* by fetal tracheal occlusion (TO). However, the changes of gene expression induced by TO remain largely unknown but could be used to further improve the clinically used prenatal treatment of this devastating malformation. Therefore, we aimed to investigate the pulmonary transcriptome changes caused by surgical induction of diaphragmatic hernia (DH) and additional TO in the fetal rabbit model. Induction of DH was associated with 378 upregulated genes compared to controls when allowing a false-discovery rate (FDR) of 0.1 and a fold change (FC) of 2. Those genes were again downregulated by consecutive TO. But DH+TO was associated with an upregulation of 157 genes compared to DH and controls. When being compared to control lungs, 106 genes were downregulated in the DH group and were not changed by TO. Therefore, the overall pattern of gene expression in DH+TO is more similar to the control group than to the DH group. In this study, we further provide a database of gene expression changes induced by surgical creation of DH and consecutive TO in the rabbit model. Future treatment strategies could be developed using this dataset. We also discuss the most relevant genes that are involved in CDH.

## Abstract (precis)

**Summary:** Rabbit fetuses with induced diaphragmatic hernia and treated with prenatal tracheal occlusion have a similar pulmonary transcriptome as unaffected controls. This study describes a valuable database of gene expressions in this model.

## INTRODUCTION

The prevalence of congenital diaphragmatic hernia (CDH) ranges between 1-4/10,000 births, which translates to 542 to 2168 children in EU-27 member states every year (2008) (Kotecha et al., 2012). It can occur either as an isolated condition, associated with other anomalies or as part of a genetic syndrome. In CDH, lung development is disturbed already from the embryonic period, but progresses because of herniation of the viscera through the defect into the chest, competing for space with the developing lungs. As a consequence, lungs of babies with CDH have fewer and less mature airway branches, a smaller cross-sectional area of pulmonary vessels, remodeled vascular architecture, and an altered vasoreactivity (Kinsella et al., 2005). At birth, this leads to respiratory insufficiency and persistent pulmonary hypertension (PPHT). This is lethal in up to 30% of cases, despite prenatal referral to high-volume centers that offer standardized neonatal care (Grushka et al., 2009; Hayakawa et al., 2013; van den Hout et al., 2011).

In countries with prenatal ultrasound (US) screening practices, >60% of cases are diagnosed at the latest by the second trimester. In isolated cases, an individualized prognosis can be made based on the degree of liver herniation and parenchymal lung size, predicting mortality and early neonatal morbidity (Cannie et al., 2008; Claus et al., 2011; Cruz-Martinez et al., 2011; Jani et al., 2007, 2009; Mayer et al., 2011; Ruano et al., 2012). The ability to prenatally identify a future non-survivor enables clinicians to propose a prenatal intervention that avoids this outcome. Today, the clinical method to stimulate lung development is fetal tracheal occlusion (TO). TO prevents egress of lung liquid, causing increased pulmonary stretch, which accelerates lung growth (DiFiore et al., 1994). This operation is currently evaluated within the framework of a randomized clinical trial, comparing fetoscopic endoluminal tracheal occlusion (FETO) to expectant management during pregnancy (Deprest et al., 2009, 2014).

Because TO still fails to save half of the fetuses with severe CDH and carries a risk for preterm delivery, there is a need for alternative prenatal strategies. Preclinical evaluation of these therapies involves the use of animal models, i.e. rodents, rabbits, and eventually lambs. Although, in all mammalians, lung development progresses through five stages (embryonic, pseudoglandular, canalicular, saccular, alveolar), this is not at the same pace (Pringle, 1986). This needs to be kept in mind when choosing an animal model in studies on lung development. For instance, rodents are ‘early’ models because CDH is induced in the embryologic phase by the administration of a teratogen (Clugston et al., 2006; Keijzer et al., 2000). In these models, nitrofen (NF) is prenatally administered at embryonic day (E)11.5 (mice) or E13.5 (rats), and effects are studied at term, when lungs are in the canalicular to saccular phase (Beurskens et al., 2007; Correia-Pinto et al., 2010). All NF-exposed pups have lung hypoplasia, half of them also the diaphragmatic defect (Keijzer et al., 2000; Rottier and Tibboel, 2005; Schittny et al., 2000). These effects are believed to be caused by interference of NF with the retinoic acid (RA) signaling pathway, which is key in lung development (Malpel et al., 2000). This pathway is also disturbed in CDH in humans (Beurskens et al., 2009).

In larger animals, hypoplasia is induced by surgical creation of a diaphragmatic defect, typically in the pseudoglandular phase. Rabbits are widely available, have low housing needs, a timed gestation and large litter size. Rabbits alveolize *in utero* as in humans (Roubliova et al., 2010). Pups with diaphragmatic hernia (DH) display both histological and functional changes, such as reduced airway and vascular development, and pathologic compliance, airway resistance, tissue damping and elastance – mimicking the clinical phenotype (Flemmer et al., 2007; Roubliova et al., 2004; Wu et al., 2000). Gene expression of a number of critical signaling molecules relevant to alveolarization, angiogenesis and regulation of vascular tone, but not to surfactant production, have been shown to be disrupted just as in humans (Vuckovic et al., 2013, 2012). However, a broader study on gene expression levels in this model has not been done so far. The use of RNA-sequencing (RNA-seq) for transcriptome analysis has become increasingly widespread with the advent of massively parallel sequencing technologies, in part owing to reductions in costs and increased throughput, and improved knowledge of non-model-organism reference genomes. Therefore, we wanted to investigate the pulmonary transcriptome after surgically induced DH creation and subsequent TO in the rabbit model. The provided gene expression database can be used to develop further treatment strategies for CDH.

## RESULTS

At harvest, there were seven surviving DH+TO fetuses [mean lung-to-body weight ratio (LBWR): 0.017; standard deviation (s.d.): 0.002; confidence interval (CI) 95%: 0.013-0.022) and seven DH fetuses (mean LBWR: 0.011; s.d.: 0.003; CI 95%: 0.003-0.018). We also took one random control for every third litter (*n*=4) (mean LBWR: 0.016; s.d.: 0.001; CI 95%: 0.013-0.018). RNA integrity (RIN) values were determined, which led to the exclusion of one DH+TO fetus. All six remaining fetuses from the DH+TO group were analyzed. We selected four (DH) fetuses displaying the best RIN values (data not shown). Principal component analysis (PCA) and unsupervised hierarchical clustering revealed that three DH+TO samples clustered together as a single homogenous group, and two DH+TO samples clustered with the DH group. Those two samples correlate with a clinical sub-group of non-responders to the TO treatment, analysis of which was not the aim of our study. The remaining DH+TO sample was considered as a statistical outlier based upon quality control (QC) correlation analysis and calculation of modified allele dosage (MAD) scores using the Array Studio software (OmicSoft, Cary, USA); hence, it was excluded from further analysis. This distinct gene expression pattern of DH+TO fetuses correlated with two patterns of lung growth, as evidenced by the LBWR, which is a pathologic measure of the degree of lung development (Table 1).

**Table 1.**
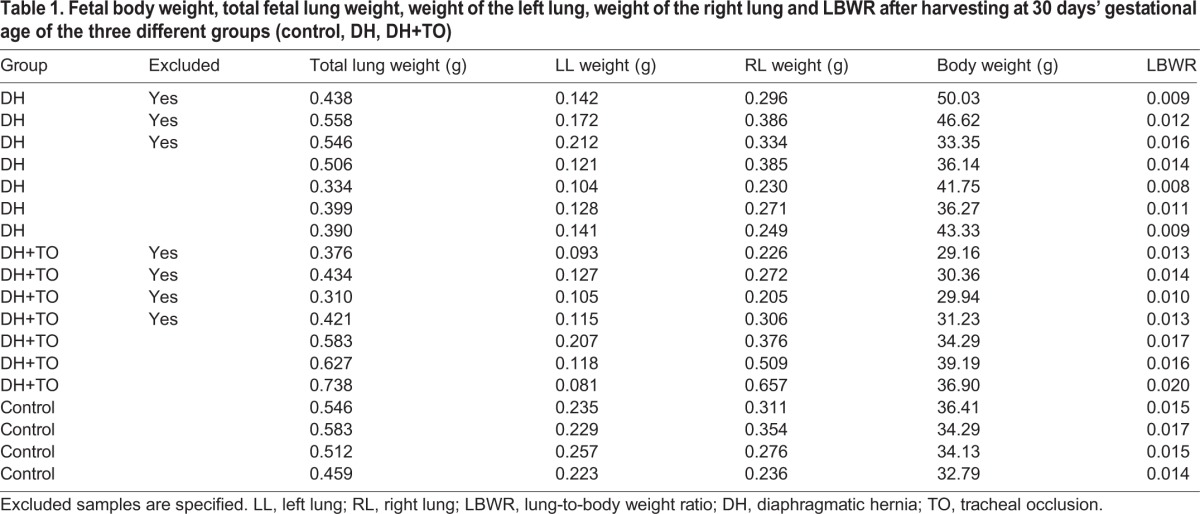
**Fetal body weight, total fetal lung weight, weight of the left lung, weight of the right lung and LBWR after harvesting at 30 days’ gestational age of the three different groups (control, DH, DH+TO)**

Statistical inference analysis was performed between the groups defined above [DH (*n*=4), DH+TO (*n*=3) and control (*n*=4)] using the general linear model function in Array Studio to calculate fold change (FC) values and false-discovery rate (FDR) values (applying the Benjamini–Hochberg procedure).

PCA and unsupervised hierarchical clustering of those dysregulated genes in any group comparison (described above) demonstrated clustering of homogenous groups for DH, DH+TO and control as expected, and revealed that DH+TO and control groups were more comparable than the DH group (Figs S1, S2).

**Fig. 1.**
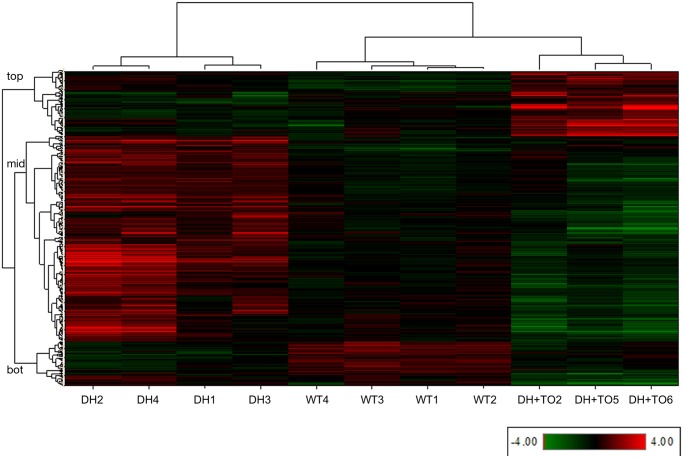
**Heat map generated by unsupervised hierarchical clustering of individual sample level data (Log2-transformed RPKM expression values per gene) for the 641 unique genes found to be dysregulated in any group comparison, demonstrating that the different groups (DH, WT and TO) cluster together.** Samples are clustered on the horizontal axis and genes on the vertical axis. Gene expression values are shown as red for high expression and green for low expression; intensity reflects the level. This reveals three large gene clusters, labeled as top, mid, bot. Information on gene names, fold changes (FC) and false-discovery rate (FDR) values for the group comparisons, and Gene Cluster and STEM profile number, are provided in Table S1. WT, control group.

The heat map generated by the unsupervised hierarchical clustering for the total of 641 unique genes found to be dysregulated (FC±2; FDR<0.1) in any group comparison is shown in Fig. 1. This reveals three large gene clusters whose expression values define the changes due to DH and after treatment with TO. The first gene cluster, containing 157 genes (Fig. 1, top) demonstrates low expression in both DH and control, and high expression in DH+TO only. This cluster represents genes whose expression is induced by TO, but which are unchanged in DH. The second, and largest, gene cluster, containing 378 genes (Fig. 1, mid) demonstrates high expression in DH only and low expression in control and DH+TO. This cluster represents genes whose expression is induced by DH, and which are reversed to lower levels by subsequent TO. The third gene cluster, containing 106 genes (Fig. 1, bot), demonstrates high expression in controls, and low expression in both DH and DH+TO. This cluster represents genes whose expression is reduced following DH, and which remain expressed at low levels even after TO.

To summarize, from the hierarchical cluster heat map shown in Fig. 1 it is apparent that the major change in gene expression (Fig. 1, gene cluster 2, mid) is upregulation of genes in DH, which are downregulated by TO towards, or beyond, levels seen in the controls (normal state) at the same developmental time point. One subset of genes is unchanged by TO and remains expressed at low levels, and one subset is highly upregulated by TO in comparison to both control and DH without TO.

The database of all dysregulated genes found in our study can be found in Table S1.

### Pathway analysis

Analysis of the dysregulated gene sets for each comparison detailed above and for each of the gene clusters defined above was performed using the IPA web application (Ingenuity Pathway Analysis, Ingenuity Systems, Redwood City, CA, USA). Of the total 641 unique genes, 560 genes were mapped within IPA to known functional genes with human, mouse or rat homologs. This gene set was used to create a protein-protein interaction (PPI) network where experimental evidence supports direct interactions (i.e. predictions and indirect interactions were not allowed), and orphan molecules were subsequently removed. The resulting network is displayed in Fig. S3, where the molecules are colored according to the gene clusters identified in Fig. 1 (blue=genes with significantly increased expression in DH compared to DH+TO and/or control; yellow=genes with significantly increased expression in DH+TO compared to DH and/or control; and green=genes with significantly decreased expression in DH and/or DH+TO in comparison to control).

The Cytoscape plugin iRegulon (Janky et al., 2014) was used to identify potential transcription factors (TFs) of interest, using the 641 genes dysregulated in any comparison as the input. iRegulon searches for enrichment of cis-regulatory TF motifs in the target gene set, and predicted FOXJ1 as a key regulatory TF in our study. The input list contains 139/641 (22%) genes that are ‘target’ genes of FOXJ1.

**Fig. 2.**
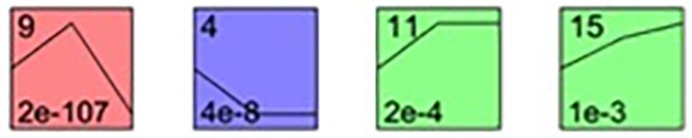
**The four significant gene profiles identified using the STEM application.** Three ‘time points’ are visible on the plots, representing changes in expression of the respective gene clusters from controls (point 1) to DH (point 2), and DH treated with TO (point 3). Profile number (see Table 2) is displayed in the top left, *P*-value bottom left. The colors are to illustrate that the changes in expression are similar in profiles 11 and 15 but different from 9 and 4.

In addition, we used the STEM application (Ernst and Bar-Joseph, 2006) to identify clusters of genes with similar changes in expression over time or, in our case, the changes observed from control to disease (DH) to treatment (DH+TO). The input was the FC values for the 641 genes significantly dysregulated in any comparison, where time point 1 is control data (i.e. FC=1), time point 2 is the CDH vs control data, and time point 3 is the TO vs control data. This identified four significant clusters containing genes with similar changes in expression profile, of which Profile 9 contained the largest number of genes and was of highest significance (Fig. 2, and Table S2).

### Real-time quantitative PCR (qPCR)

PCR analysis of ten of the 12 analyzed genes yielded results that were in agreement with RNA-seq data (Fig. S4). For eight genes, the differential expression between groups was concordant with the RNA-seq data (*P*<0.05, Mann–Whitney *U* test). However, qPCR did not show a significant increase of *RFX3* and *LRRQI1* in the DH group compared to control (Fig. S5). For *BMPR2* and *PDE5A*, qPCR failed to show any difference between DH and wild type. This distinct gene expression pattern of DH+T fetuses correlated with two patterns of lung growth, as evidenced by the LBWR, which is a pathologic measure of the degree of lung development. However, *PDE5A* was significantly downregulated in the TO group.

## DISCUSSION

In this study, we describe for the first time the pulmonary transcriptome analysis of specimens obtained in a rabbit model for pulmonary hypoplasia. The latter was induced by creating a diaphragmatic defect during the pseudoglandular phase. Conversely, forced lung growth was induced by fetal TO at the transition of the canalicular to saccular phase. We found that the largest group of genes that were significantly dysregulated were 378 genes that were both upregulated by DH creation and downregulated by TO to a level similar to that of controls. Furthermore, this study gives a database of genes that are significantly influenced by DH creation and consecutive TO (Table S1). This database can be used for further understanding of the disease process and development of treatment modalities for CDH. Below, we discuss some of the most relevant genes that we found were dysregulated.

### Relation of findings to previous gene expression analytical experiments in other models of CDH and/or TO

Many studies have documented expression changes for numerous genes in association with CDH and, to a lesser extent, also the effects of TO, all of this in various animal models of CDH. This is typically done by using PCR for selected genes, or using broader arrays, at least for experiments done in (NF-exposed) rodents, a species in which molecular tools are abundantly available. Using a more modern technique such as RNA-seq, one can now also document and screen for changes in gene expression in relevant animal models for pulmonary hypoplasia and induced lung growth, even if the genome has not been completely identified. We herein used this technology in rabbits, and studied dysregulations associated with pulmonary hypoplasia and TO. The latter is done to force lung growth, which has been abundantly demonstrated in several animal models of CDH using other tools (Khan et al., 2007). In this experiment, we confirmed the effects of TO on cell proliferation. MKI67 is a key cell proliferation marker and was significantly upregulated by TO. The effect of TO was in proportion to the pre-existing lung size (FC 3.6056, FDR 0.0874; TO vs control), a phenomenon already clinically demonstrated (Peralta et al., 2008). Other genes related to the mechanisms of TO in previous studies were identified. To name only one, *AQP4* was upregulated in DH (FC 2.3562, FDR 0.018) yet went down to a normal level after TO (FC −2.7286, FDR 0.0106). AQP4 is a transport molecule that has previously been shown to be downregulated by stretch force on fetal lung AT2 cells *in vitro* (Wang et al., 2006).

TO is, however, in essence performed to enhance alveologenesis and reverse vascular changes that are typical of this disease. We observed dysregulation of connective tissue growth factor (*CTGF*). CTGF indeed enhances alveologenesis and microvascularization during late lung development (Hall-Glenn and Lyons, 2011). CTGF was earlier shown to be upregulated by TO in the saccular phase of E21 rats (Burgos et al., 2010; Mesas-Burgos et al., 2009). In line with that, *CTGF* was, in our rabbits, also firmly upregulated by TO (FC 2.8135, FDR 0.0151). The same dysregulation was observed for a number of other genes that have been previously associated with DH and TO. *BMPR2* expression was increased in DH (FC 1.9265, FDR 0.0228) and went down after TO (FC −2.7956, FDR 0.0097). This gene encodes a member of the bone morphogenetic protein (BMP) receptor family of transmembrane serine/threonine kinases and the ligands of this receptor are the BMPs, which are members of the TGF-β superfamily and are involved in embryogenesis. Disruption of BMPR2 activity and downstream signaling has been demonstrated in the NF rodent model of CDH (Gosemann et al., 2013; Makanga et al., 2013). BMPR2 plays a key role in pulmonary vasculogenesis during the late stages of fetal lung development and is essential for control of endothelial and smooth-muscle cell proliferation. Dysfunction of BMPR2 and downstream signaling have been shown to disturb the crucial balance of proliferation of smooth muscle cells, contributing to the pathogenesis of human and experimental pulmonary hypoplasia (Gosemann et al., 2013; Makanga et al., 2013). In line with this was downregulation of BMP7 in DH, yet significant upregulation by TO (FC 2.311, FDR 0.0163). BMP7 was reportedly downregulated in the lungs of the NF rodent (Makanga et al., 2013).

We were, however, mainly interested in the RNA-seq technology because it has the potential to identify new targets that are involved in the condition and that can respond to prenatal intervention. One example is that DH in our rabbits was associated with a significant increase in *PDE5A* gene expression. The latter has been well documented in rodents and sheep, and it has been tied to the postnatal problem of pulmonary hypertension (Noori et al., 2007). The interesting observation here is that TO dramatically reduced *PDE5A* gene expression (FC −2.49; FDR 0.0097). *PDE5A* gene expression might also be altered by an alternative prenatal intervention, i.e. the transplacental administration of drugs. Sildenafil, which is already used to treat PPHT of the newborn (Noori et al., 2007), was recently shown to effectively inhibit cyclic guanosine monophosphate (cGMP)-specific phosphodiesterase type 5 *in utero* (Luong et al., 2011). In the latter experiment, NF rodents were used, and we confirmed a similar effect in rabbits, and others in the fetal lamb (Luong et al., 2011; Russo et al., 2015; Shue et al., 2014).

### Future application: single cell-type populations

When starting from our RNA-seq data, the Cytoscape plugin iRegulon predicted that over 20% of dysregulated genes were target genes of FOXJ1. FOXJ1 is a marker for ciliated cells; it is actually involved as a transcription factor in ciliogenesis (Yu et al., 2008). To our knowledge, it has so far not been named in DH. Our experiments demonstrate that DH rabbit pups display upregulation of *FOXJ1*, whereas it was downregulated after TO. Other ciliated-cell marker genes (*CCDC19*, *SPAG17*, *CCDC39*, *LRRIQ1*, *EFHC1*, *LRRC23*, *TTC18*, *WDR16*, *FANK1*, *ENKUR*, *CCDC113*) were also upregulated in DH lungs compared to controls, and downregulated by TO to levels lower than in controls. These findings point to an increased number of ciliated cells in DH lungs, which is reversed by TO. Loss of ciliated cells after TO has been previously described in the upper conducting airways in fetal lambs with CDH, although that study did not quantify changes in the more distant airways (Deprest et al., 2000). The study of effects of DH and TO on the variety of individual lung cell types can benefit from modern technology such as single-cell RNA-seq (Treutlein et al., 2014). Those investigators applied this method to the developing distal lung epithelium in the mouse, which revealed a number of putative markers of ciliated cells, Clara cells, alveolar type 1 (AT1) cells and AT2 cells. A number of genes in our data are also present within the top 30 putative markers for each lung cell type (both known and novel) previously identified in the Treutlein et al. (2014) study (Table 2). For instance, the Clara cell markers *SCGB1A1*, *PPAP2B* and *KDR* (*VEGFR2*) were also upregulated in DH, and subsequently downregulated after TO. These findings are consistent with findings in rodents (Asabe et al., 1998; Santos et al., 2007).

**Table 2.**
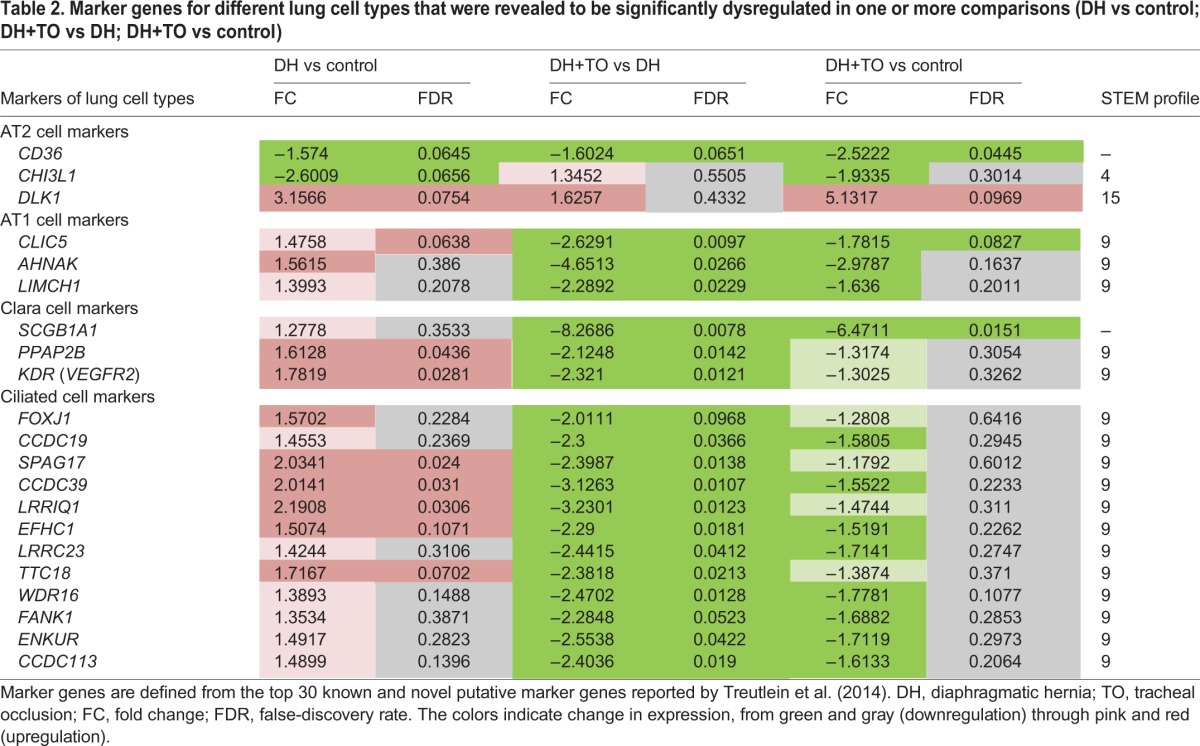
**Marker genes for different lung cell types that were revealed to be significantly dysregulated in one or more comparisons (DH vs control; DH+TO vs DH; DH+TO vs control)**

### Discordant findings and limitations

The above is a simplified interpretation and biased presentation of our results. First, we identified a number of discrepancies in results between rabbits and other models. For example, *AQP4*, *TGFBR3*, *EPAS1* and *TGFB-I* were upregulated in rabbits with CDH, whereas they were downregulated in the NF rat. In rodents, DH is induced early in gestation by a teratogen interfering directly with the RA pathway, whereas, in rabbits, a surgical defect is created beyond mid-gestation. This means that, in both models, different mechanisms or pathways are involved. Therefore, prenatal interventions might have different effects. For instance, maternal retinoic acid administration in rabbits does not affect the gross anatomical, morphological or proliferative characteristics (Gallot et al., 2008), whereas it rescues lungs in NF rats (Montedonico et al., 2006). Next to that there are also large differences between the time points in gestation and/or lung development at which prenatal interventions are done.

Actually, the largest effect measured was that for WNT inhibitor factor 1 (*WIF-1*) gene expression [up in DH (FC 8.8107, FDR 0.0153) and down after TO (FC −4.1432, FDR 0.0272)]. However, *WIF-1* downregulation was previously reported in the NF rodent lungs during the saccular stage of lung development (Fujiwara et al., 2012). WNT signaling plays an essential role in embryonic development. *WIF1* is a target gene of SMAD1 in the developing lung epithelial cells and acts to inhibit WNT proteins (Xu et al., 2011). SMAD1 plays a key role in organogenesis, including in lung development and maturation, and *SMAD1*-knockout mice display reduced sacculation, which is an important feature of pulmonary hypoplasia (References?). How this needs to be tied together with our rabbit data remains unclear.

In addition, we observed changes that were different from what was previously described in clinical specimens. One example is that of the endothelin receptors EDNRA and EDNRB, which play a complex role in vascular tone. They are overexpressed in the thickened media of the pulmonary arteries of newborns with CDH (de Lagausie et al., 2005), as well as in the NF rodent model (Dingemann et al., 2010). Binding of endothelin 1 to its receptor EDNRA on pulmonary artery smooth-muscle cells results in vasoconstriction, whereas binding to the receptor EDNRB, present on endothelial cells, results in vasodilation mediated by endogenous nitric oxide. In our rabbit model, however, *EDNRB* was not differently expressed in DH as compared to controls.

We also observed discrepancies between the effects of DH and TO within the rabbit model. For instance c-fos induced growth factor [*FIGF*; also known as vascular endothelial growth factor D (*VEGFD*)] was downregulated in CDH as well as following TO. This is a member of the PDGF/VEGF family, and activates VEGFR2 and VEGFR3 receptors. Because it was not differentially expressed in the controls, we assume it was not involved. Conversely, VEGFR2 was upregulated in DH (FC 1.7819, FDR 0.0281) and down to normal levels after TO (FC −2.321, FDR 0.0121), which demonstrates the complex interactions of these factors.

We further had a number of discrepancies between RNA-seq and qPCR, which we touched upon already when reporting on results for *RFX3*, *LRRQL1*, *BMPR2* and *PDE5A*. Such discrepancies have been observed in previous reports (Duressa et al., 2013; Shi and He, 2014). This lack of concordance might be due to the poor knowledge of the rabbit genome and the absence of any single nucleotide polymorphism (SNP) database, which could result in poor primer design for certain genes.

Additionally, we acknowledge several other limitations to our study. The sample size is small yet it matches financial limitations and is in line with the sample size of other studies analyzing the gene expression changes by TO in DH. Another is that the study of gene expression in whole lung samples will not detect changes occurring in individual cell types or different areas along the airways. These add to the generic limitations of translational studies in animal disease models, of which the clinical relevance remains uncertain. Another limitation of the study is that the surgical model does not fully recapitulate the etiology of the disease.

In conclusion, we first describe the pattern of dysregulated pulmonary gene expression in lungs of fetal rabbits with surgically induced DH. Interestingly, TO (tracheal occlusion), which is currently investigated as a prenatal surgical method to reverse pulmonary hypoplasia, is associated with a gene expression pattern that is comparable to what was observed in littermates with normal lung development.

## MATERIALS AND METHODS

### Surgery

The animal experiments were approved by the Ethics Committee of the Faculty of Medicine of the KU Leuven and all animals were treated according to current guidelines on animal wellbeing. In total, 12 does of New Zealand white rabbits (*Oryctolagus cuniculus*) were operated on and premedicated, after weight estimation, with ketamine 50 mg/kg body weight (Ketamine 1000 CEVA^®^; CEVA Santé Animale, Brussels, Belgium), xylazine 6 mg/kg body weight (Vexylan^®^; CEVA Santé Animale) and buprenorphine 0.03 mg/kg body weight (Vetergesic^®^; Reckitt Benckiser Healthcare, Brussels, Belgium), all injected intramuscularly. General anesthesia was maintained using isoflurane 1.5% (Isoba^®^ Vet; Abbott Laboratories Ltd, Queensborough, Kent, UK) in oxygen at 1 liter/min via a facemask. Maternal heart rate and oxygen saturation were monitored with a pulse oxymeter (Nellcor^®^ N-20P; Nellcor Inc., Haasrode, Belgium). Physiologic body temperature was maintained by a heating pad. The doe was placed in the supine position, the abdominal wall was shaved and disinfected with povidone iodine (Isobetadine^®^; Asta Medica, Brussels, Belgium) and covered with sterile drapes. Aseptic conditions were maintained throughout all surgical procedures. We operated on two fetuses in each doe, located in the middle of the left or right uterine horn. First diaphragmatic hernia (DH) was created at 23 days gestational age (GA). A second operation was performed at 28 days GA on the previously operated fetuses: either tracheal occlusion (DH+TO group), or sham neck dissection and skin closure (DH group). The latter is performed to include the effects of the fetal surgery *per se*. Before and after surgery, animals were housed in separate cages at normal room temperature and daylight, with free access to food and water. At 30 days GA, the does were euthanized with an intravenous bolus of a mixture of embutramide 200 mg, mebezonium 50 mg and tetracaine hydrochloride 5 mg (0.3 ml/kg T61; Marion Roussel Hoechst, Brussels, Belgium) after previous premedication as described above. All operated fetuses as well as one control unoperated littermate (control group) were euthanized *in utero* and harvested by cesarean section to obtain non-ventilated lungs.

### Harvesting and sample preparation

During necropsy, the fetus and its lungs were weighed on a scale measuring accurately up to 0.001 g (HF 2000; A&D Instruments, Haasrode, Belgium) and the lung-to-body weight ratio (LBWR) was calculated.

Lung development is reflected by the LBWR and pathologists have defined critical cut-offs for pulmonary hypoplasia. We minimized contamination of the pulmonary tissue with other sources of DNA/RNA by flushing the lungs with saline by vascular access through the right ventricle of the fetal heart. The parenchymal lung tissue, i.e. without the trachea, was stored in RNAlater (Qiagen Benelux B.V., Venlo NL) at −4°C immediately.

### RNA isolation

RNA isolation was performed on the entire left lung within 4 h of harvesting using the RNeasy mini kit (Qiagen Benelux B.V., Venlo NL). Tissue lysis and homogenization was performed in 1200 µl Buffer RLT using the TissueLyser system (Qiagen Benelux B.V., Venlo, The Netherlands). Following tissue disruption and homogenization, samples were centrifuged for 3 min at 8764 ***g*** (14,000 rpm) in a benchtop microcentrifuge. Lysate was transferred to fresh tubes and an equal volume of 70% ethanol was added. 600 µl of sample was added twice to a spin column, with two RNeasy spin columns used per sample. Following wash steps, RNA was eluted in 50 µl RNAse-free H_2_O.

Total RNA quantification was performed using the Nanodrop 1000 spectrophotometer (Thermo Scientific, Aalst, Belgium). RNA integrity was assessed using the RNA 6000 Nano Kit and the Bioanalyser (Agilent Technologies, Diegem, Belgium) according to the manufacturer's recommendations.

### RNA-seq library preparation

One µg of total RNA was used as input material for sequencing library preparation, which was performed with the TruSeq RNA Library Preparation Kit (Illumina, Eindhoven, NL) according to the manufacturer's protocol. Fragmentation was performed for 6 min. Eight PCR cycles were used for the PCR enrichment step. Samples were indexed to allow for multiplexing. Sequencing libraries were quantified using the Qubit fluorometer (Life Technologies Europe B.V., Ghent, Belgium). Library quality and size range was assessed using the Bioanalyser (Agilent Technologies) with the DNA 1000 Kit (Agilent Technologies) according to the manufacturer's recommendations.

### RNA-seq

Individual libraries were diluted to a final concentration of 2 nM and pooled for sequencing. Pooled libraries were sequenced in a single lane of an Illumina HiSeq2000 flow cell generating single end 50 bp reads. At least 10 million reads were obtained for all samples (range 12- to 32-million reads).

### Data analysis

Fastq files were mapped to the rabbit genome and transcriptome (OryCun2.0 retrieved from Ensembl, release 69) with the Array Studio software (OmicSoft, Cary, NC, USA) using default parameters for single short read data. Between 72% and 78% of reads mapped uniquely to the reference genome and/or transcriptome, and a further 6% to 8% of reads mapped non-uniquely. Between 16% and 18% of reads were unmapped. Expression values were calculated per gene, normalized to ‘reads per kilobase per million reads’ (RPKM) values as previously described (Mortazavi et al., 2008). Data were filtered to remove those genes with expression of <1 RPKM in all samples. The RPKM expression values for the remaining 11,267 genes were subsequently Log2 transformed for downstream PCA, hierarchical clustering, and for the subsequent calculation of fold changes (FC) by statistical inference analysis. All data are made available via ArrayExpress (accession number: E-MTAB-3452).

### Hierarchical clustering

Hierarchical clustering was performed using the Array Studio software application, selecting ‘Complete’ for link option, and ‘Correlation’ for distance option. The ‘Correlation’ setting is also named ‘Centered Pearson’ 1−corr(*x*, *y*), and the source algorithms are further described in http://cran.r-project.org/web/packages/amap/amap.pdf. Clustering is based upon the Log-transformed RPKM gene expression values.

### Downstream analysis

Downstream pathway analysis and creation of networks was performed using the IPA software application (Ingenuity Systems, Redwood City, CA, USA). The iRegulon plugin was used with the Cytoscape analysis software (http://www.cytoscape.org/) for prediction of upstream transcription factors of interest (Janky et al., 2014). The short time-series expression miner (STEM) application (Ernst and Bar-Joseph, 2006) was used to identify gene clusters with similar changes in expression between control and CDH, and following TO.

### Real-time quantitative PCR (qPCR)

In order to confirm RNA-seq findings, we selected for qPCR (quantitative polymerase chain reaction) 12 genes with FC>2 or FC<(−2) and the housekeeping genes *SDHA*, *TOP1* and *GAPDH*, which were previously shown to be among the most stable genes in the CDH rabbit model and after TO (Vuckovic et al., 2013). PCR primers were designed using Primer 3 software and Primer-BLAST. The absence of secondary structure was checked by the Vector NTI program. All the primers were synthesized by Integrated DNA Technologies.

For a given gene, all samples were analyzed in the same qPCR run. 10-µl reactions were prepared using 2× LightCycler 480 SYBR Green I Master (Roche Applied Science) with 2 µl of a 1:10 cDNA dilution and a final concentration of 300 nM of each primer. Data were analyzed using the LightCycler 480 software (Roche Applied Science). The results were quantified using the comparative threshold cycle method as described by Livak and Schmittgen (2001). In addition, serial dilutions were used to create standard curves for relative quantification and the expression of each gene was normalized to at least two of the three housekeeping genes' expression. The sequences and the different primers and the corresponding PCR efficiencies are provided in Table S3.

## References

[DMM021626C1] Asabe K., Tsuji K., Handa N., Kajiwara M. and Suita S. (1998). Expression of clara cell 10-kDa protein (CC10) in congenital diaphragmatic hernia. *Pediatr. Surg. Int.* 14, 36-39. 10.1007/s003830050430 9880692

[DMM021626C2] Beurskens N., Klaassens M., Rottier R., de Klein A. and Tibboel D. (2007). Linking animal models to human congenital diaphragmatic hernia. *Birth Defects Res. A Clin. Mol. Teratol.* 79, 565-572. 10.1002/bdra.20370 17469205

[DMM021626C3] Beurskens L. W. J. E., Tibboel D. and Steegers-Theunissen R. P. M. (2009). Role of nutrition, lifestyle factors, and genes in the pathogenesis of congenital diaphragmatic hernia: human and animal studies. *Nutr. Rev.* 67, 719-730. 10.1111/j.1753-4887.2009.00247.x 19941617

[DMM021626C4] Burgos C. M., Nord M., Roos A., Didon L., Eklof A.-C. and Frenckner B. (2010). Connective tissue growth factor expression pattern in lung development. *Exp. Lung Res.* 36, 441-450. 10.3109/01902141003714056 20939759

[DMM021626C5] Cannie M., Jani J., Chaffiotte C., Vaast P., Deruelle P., Houfflin-Debarge V., Dymarkowski S. and Deprest J. (2008). Quantification of intrathoracic liver herniation by magnetic resonance imaging and prediction of postnatal survival in fetuses with congenital diaphragmatic hernia. *Ultrasound Obstet. Gynecol.* 32, 627-632. 10.1002/uog.6146 18792415

[DMM021626C6] Claus F., Sandaite I., DeKoninck P., Moreno O., Cruz Martinez R., Van Mieghem T., Gucciardo L., Richter J., Michielsen K., Decraene J. et al. (2011). Prenatal anatomical imaging in fetuses with congenital diaphragmatic hernia. *Fetal Diagn. Ther.* 29, 88-100. 10.1159/000320605 21063073

[DMM021626C7] Clugston R. D., Klattig J., Englert C., Clagett-Dame M., Martinovic J., Benachi A. and Greer J. J. (2006). Teratogen-induced, dietary and genetic models of congenital diaphragmatic hernia share a common mechanism of pathogenesis. *Am. J. Pathol.* 169, 1541-1549. 10.2353/ajpath.2006.060445 17071579 PMC1780206

[DMM021626C8] Correia-Pinto J., Gonzaga S., Huang Y. and Rottier R. (2010). Congenital lung lesions--underlying molecular mechanisms. *Semin. Pediatr. Surg.* 19, 171-179. 10.1053/j.sempedsurg.2010.03.003 20610189

[DMM021626C9] Cruz-Martinez R., Hernandez-Andrade E., Moreno-Alvarez O., Done E., Deprest J. and Gratacos E. (2011). Prognostic value of pulmonary Doppler to predict response to tracheal occlusion in fetuses with congenital diaphragmatic hernia. *Fetal Diagn. Ther.* 29, 18-24. 10.1159/000320249 20881369

[DMM021626C10] de Lagausie P., de Buys-Roessingh A., Ferkdadji L., Saada J., Aisenfisz S., Martinez-Vinson C., Fund X., Cayuela J. M., Peuchmaur M., Mercier J. C. et al. (2005). Endothelin receptor expression in human lungs of newborns with congenital diaphragmatic hernia. *J. Pathol.* 205, 112-118. 10.1002/path.1677 15546126

[DMM021626C11] Deprest J. A. M., Evrard V. A., Verbeken E. K., Perales A. J., Delaere P. R., Lerut T. E. and Flageole H. (2000). Tracheal side effects of endoscopic balloon tracheal occlusion in the fetal lamb model. *Eur. J. Obstet. Gynecol. Reprod. Biol.* 92, 119-126. 10.1016/S0301-2115(00)00435-8 10986445

[DMM021626C12] Deprest J. A., Hyett J. A., Flake A. W., Nicolaides K. and Gratacos E. (2009). Current controversies in prenatal diagnosis 4: should fetal surgery be done in all cases of severe diaphragmatic hernia? *Prenat. Diagn.* 29, 15-19. 10.1002/pd.2108 19125386

[DMM021626C13] Deprest J., Brady P., Nicolaides K., Benachi A., Berg C., Vermeesch J., Gardener G. and Gratacos E. (2014). Prenatal management of the fetus with isolated congenital diaphragmatic hernia in the era of the TOTAL trial. *Semin. Fetal Neonatal. Med.* 19, 338-348. 10.1016/j.siny.2014.09.006 25447987

[DMM021626C14] DiFiore J. W., Fauza D. O., Slavin R., Peters C. A., Fackler J. C. and Wilson J. M. (1994). Experimental fetal tracheal ligation reverses the structural and physiological effects of pulmonary hypoplasia in congenital diaphragmatic hernia. *J. Pediatr. Surg.* 29, 248-256; discussion 256–257 10.1016/0022-3468(94)90328-X 8176601

[DMM021626C15] Dingemann J., Doi T., Ruttenstock E. and Puri P. (2010). Upregulation of endothelin receptors A and B in the nitrofen induced hypoplastic lung occurs early in gestation. *Pediatr. Surg. Int.* 26, 65-69. 10.1007/s00383-009-2514-8 19851775

[DMM021626C16] Duressa D., Anchieta A., Chen D., Klimes A., Garcia-Pedrajas M. D., Dobinson K. F. and Klosterman S. J. (2013). RNA-seq analyses of gene expression in the microsclerotia of Verticillium dahliae. *BMC Genomics* 14, 607 10.1186/1471-2164-14-607 24015849 PMC3852263

[DMM021626C17] Ernst J. and Bar-Joseph Z. (2006). STEM: a tool for the analysis of short time series gene expression data. *BMC Bioinformatics* 7, 191 10.1186/1471-2105-7-191 16597342 PMC1456994

[DMM021626C18] Flemmer A. W., Jani J. C., Bergmann F., Muensterer O. J., Gallot D., Hajek K., Sugawara J., Till H. and Deprest J. A. (2007). Lung tissue mechanics predict lung hypoplasia in a rabbit model for congenital diaphragmatic hernia. *Pediatr. Pulmonol.* 42, 505-512. 10.1002/ppul.20618 17469148

[DMM021626C19] Fujiwara N., Doi T., Gosemann J.-H., Kutasy B., Friedmacher F. and Puri P. (2012). Smad1 and WIF1 genes are downregulated during saccular stage of lung development in the nitrofen rat model. *Pediatr. Surg. Int.* 28, 189-193. 10.1007/s00383-011-2987-0 21986617

[DMM021626C20] Gallot D., Coste K., Jani J., Roubliova X., Marceau G., Velemir L., Verheyen A., Lemery D., Sapin V. and Deprest J. (2008). Effects of maternal retinoic acid administration in a congenital diaphragmatic hernia rabbit model. *Pediatr. Pulmonol.* 43, 594-603. 10.1002/ppul.20829 18435480

[DMM021626C21] Gosemann J.-H., Friedmacher F., Fujiwara N., Alvarez L. A. J., Corcionivoschi N. and Puri P. (2013). Disruption of the bone morphogenetic protein receptor 2 pathway in nitrofen-induced congenital diaphragmatic hernia. *Birth Defects Res. B Dev. Reprod. Toxicol.* 98, 304-309. 10.1002/bdrb.21065 23780850

[DMM021626C22] Grushka J. R., Laberge J.-M., Puligandla P. and Skarsgard E. D. (2009). Effect of hospital case volume on outcome in congenital diaphragmatic hernia: the experience of the Canadian Pediatric Surgery Network. *J. Pediatr. Surg.* 44, 873-876. 10.1016/j.jpedsurg.2009.01.023 19433160

[DMM021626C501] Hall-Glenn F. and Lyons K. M. (2011). Roles for CCN2 in normal physiological processes. *Cell. Mol. Life. Sci.* 68, 3209-3217. 10.1007/s00018-011-0782-7 21858450 PMC3670951

[DMM021626C23] Hayakawa M., Ito M., Hattori T., Kanamori Y., Okuyama H., Inamura N., Takahashi S., Nagata K., Taguchi T., Usui N. et al. (2013). Effect of hospital volume on the mortality of congenital diaphragmatic hernia in Japan. *Pediatr. Int.* 55, 190-196. 10.1111/ped.12059 23360371

[DMM021626C24] Jani J., Nicolaides K. H., Keller R. L., Benachi A., Peralta C. F. A., Favre R., Moreno O., Tibboel D., Lipitz S., Eggink A. et al. (2007). Observed to expected lung area to head circumference ratio in the prediction of survival in fetuses with isolated diaphragmatic hernia. *Ultrasound Obstet. Gynecol.* 30, 67-71. 10.1002/uog.4052 17587219

[DMM021626C25] Jani J. C., Benachi A., Nicolaides K. H., Allegaert K., Gratacós E., Mazkereth R., Matis J., Tibboel D., Van Heijst A., Storme L. et al. (2009). Prenatal prediction of neonatal morbidity in survivors with congenital diaphragmatic hernia: a multicenter study. *Ultrasound Obstet. Gynecol.* 33, 64-69. 10.1002/uog.6141 18844275

[DMM021626C26] Janky R., Verfaillie A., Imrichová H., Van de Sande B., Standaert L., Christiaens V., Hulselmans G., Herten K., Naval Sanchez M., Potier D. et al. (2014). iRegulon: from a gene list to a gene regulatory network using large motif and track collections. *PLoS Comput. Biol.* 10, e1003731 10.1371/journal.pcbi.1003731 25058159 PMC4109854

[DMM021626C27] Keijzer R., Liu J., Deimling J., Tibboel D. and Post M. (2000). Dual-hit hypothesis explains pulmonary hypoplasia in the nitrofen model of congenital diaphragmatic hernia. *Am. J. Pathol.* 156, 1299-1306. 10.1016/S0002-9440(10)65000-6 10751355 PMC1876880

[DMM021626C28] Khan P. A., Cloutier M. and Piedboeuf B. (2007). Tracheal occlusion: a review of obstructing fetal lungs to make them grow and mature. *Am. J. Med. Genet. C Semin. Med. Genet.* 145C, 125-138. 10.1002/ajmg.c.30127 17436297

[DMM021626C29] Kinsella J. P., Ivy D. D. and Abman S. H. (2005). Pulmonary vasodilator therapy in congenital diaphragmatic hernia: acute, late, and chronic pulmonary hypertension. *Semin. Perinatol.* 29, 123-128. 10.1053/j.semperi.2005.04.008 16052736

[DMM021626C30] Kotecha S., Barbato A., Bush A., Claus F., Davenport M., Delacourt C., Deprest J., Eber E., Frenckner B., Greenough A. et al. (2012). Congenital diaphragmatic hernia. *Eur. Respir. J.* 39, 820-829. 10.1183/09031936.00066511 22034651

[DMM021626C31] Livak K. J. and Schmittgen T. D. (2001). Analysis of relative gene expression data using real-time quantitative PCR and the 2(−Delta Delta C(T)) Method. *Methods* 25, 402-408. 10.1006/meth.2001.1262 11846609

[DMM021626C502] Luong C., Rey-Perra J., Vadivel A., Gilmour G., Sauve Y., Koonen D., Walker D., Todd K. G., Gressens P., Kassiri Z. et al. (2011). Antenatal sildenafil treatment attenuates pulmonary hypertension in experimental congenital diaphragmatic hernia. *Circulation* 123, 2120-2131. 10.1161/CIRCULATIONAHA.108.845909 21537000

[DMM021626C32] Makanga M., Dewachter C., Maruyama H., Vuckovic A., Rondelet B., Naeije R. and Dewachter L. (2013). Downregulated bone morphogenetic protein signaling in nitrofen-induced congenital diaphragmatic hernia. *Pediatr. Surg. Int.* 29, 823-834. 10.1007/s00383-013-3340-6 23832098

[DMM021626C33] Malpel S., Mendelsohn C. and Cardoso W. V. (2000). Regulation of retinoic acid signaling during lung morphogenesis. *Development* 127, 3057-3067. 10862743 10.1242/dev.127.14.3057

[DMM021626C34] Mayer S., Klaritsch P., Petersen S., Done E., Sandaite I., Till H., Claus F. and Deprest J. A. (2011). The correlation between lung volume and liver herniation measurements by fetal MRI in isolated congenital diaphragmatic hernia: a systematic review and meta-analysis of observational studies. *Prenat. Diagn.* 31, 1086-1096. 10.1002/pd.2839 21915885

[DMM021626C35] Mesas-Burgos C., Nord M., Didon L., Eklöf A.-C. and Frenckner B. (2009). Gene expression analysis after prenatal tracheal ligation in fetal rat as a model of stimulated lung growth. *J. Pediatr. Surg.* 44, 720-728. 10.1016/j.jpedsurg.2008.06.020 19361631

[DMM021626C36] Montedonico S., Nakazawa N. and Puri P. (2006). Retinoic acid rescues lung hypoplasia in nitrofen-induced hypoplastic foetal rat lung explants. *Pediatr. Surg. Int.* 22, 2-8. 10.1007/s00383-005-1571-x 16284794

[DMM021626C37] Mortazavi A., Williams B. A., McCue K., Schaeffer L. and Wold B. (2008). Mapping and quantifying mammalian transcriptomes by RNA-Seq. *Nat. Methods* 5, 621-628. 10.1038/nmeth.1226 18516045 PMC13303166

[DMM021626C38] Noori S., Friedlich P., Wong P., Garingo A. and Seri I. (2007). Cardiovascular effects of sildenafil in neonates and infants with congenital diaphragmatic hernia and pulmonary hypertension. *Neonatology* 91, 92-100. 10.1159/000097125 17344658

[DMM021626C39] Peralta C. F. A., Jani J. C., Van Schoubroeck D., Nicolaides K. H. and Deprest J. A. (2008). Fetal lung volume after endoscopic tracheal occlusion in the prediction of postnatal outcome. *Am. J. Obstet. Gynecol.* 198, 60.e1–60.e5. 10.1016/j.ajog.2007.05.034 17826727

[DMM021626C40] Pringle K. C. (1986). Human fetal lung development and related animal models. *Clin. Obstet. Gynecol.* 29, 502-513. 10.1097/00003081-198609000-00006 3757332

[DMM021626C41] Rottier R. and Tibboel D. (2005). Fetal lung and diaphragm development in congenital diaphragmatic hernia. *Semin. Perinatol.* 29, 86-93. 10.1053/j.semperi.2005.04.004 16050526

[DMM021626C42] Roubliova X., Verbeken E., Wu J., Yamamoto H., Lerut T., Tibboel D. and Deprest J. (2004). Pulmonary vascular morphology in a fetal rabbit model for congenital diaphragmatic hernia. *J. Pediatr. Surg.* 39, 1066-1072. 10.1016/j.jpedsurg.2004.03.049 15213901

[DMM021626C43] Roubliova X. I., Deprest J. A., Biard J. M., Ophalvens L., Gallot D., Jani J. C., Van de Ven C. P., Tibboel D. and Verbeken E. K. (2010). Morphologic changes and methodological issues in the rabbit experimental model for diaphragmatic hernia. *Histol. Histopathol.* 25, 1105-1116. 20607652 10.14670/HH-25.1105

[DMM021626C44] Ruano R., Takashi E., da Silva M. M., Campos J. A. D. B., Tannuri U. and Zugaib M. (2012). Prediction and probability of neonatal outcome in isolated congenital diaphragmatic hernia using multiple ultrasound parameters. *Ultrasound Obstet. Gynecol.* 39, 42-49. 10.1002/uog.10095 21898639

[DMM021626C503] Russo F., MiYague A. H., Eastwood P., DeKoninck P., Van de Velde G., Allegaert K., Himmelreich U., Toelen J., Vergani P. and Deprest J. (2015). Transplacental sildenafil rescues vascular and airway morphometry in the rabbit model of congenital diaphragmatic hernia. In *35th Annual Meeting of the Society for Maternal-Fetal Medicine*, vol. 212, pp. S44-S45. San Diego 10.1016/j.ajog.2014.10.109

[DMM021626C45] Santos M., Nogueira-Silva C., Baptista M. J., Soares-Fernandes J., Moura R. S. and Correia-Pinto J. (2007). Pulmonary epithelial cell differentiation in the nitrofen-induced congenital diaphragmatic hernia. *J. Pediatr. Surg.* 42, 1231-1237. 10.1016/j.jpedsurg.2007.02.014 17618886

[DMM021626C46] Schittny J. C., Miserocchi G. and Sparrow M. P. (2000). Spontaneous peristaltic airway contractions propel lung liquid through the bronchial tree of intact and fetal lung explants. *Am. J. Respir. Cell Mol. Biol.* 23, 11-18. 10.1165/ajrcmb.23.1.3926 10873148

[DMM021626C47] Shi Y. and He M. (2014). Differential gene expression identified by RNA-Seq and qPCR in two sizes of pearl oyster (Pinctada fucata). *Gene* 538, 313-322. 10.1016/j.gene.2014.01.031 24440293

[DMM021626C504] Shue E. H., Schecter S. C., Gong W., Etemadi M., Johengen M., Iqbal C., Derderian S. C., Oishi P., Fineman J. R. and Miniati D. (2014). Antenatal maternally-administered phosphodiesterase type 5 inhibitors normalize eNOS expression in the fetal lamb model of congenital diaphragmatic hernia. *J. Pediatr. Surg.* 49, 39-45; discussion 45 10.1016/j.jpedsurg.2013.09.024 24439578 PMC3896891

[DMM021626C48] Treutlein B., Brownfield D. G., Wu A. R., Neff N. F., Mantalas G. L., Espinoza F. H., Desai T. J., Krasnow M. A. and Quake S. R. (2014). Reconstructing lineage hierarchies of the distal lung epithelium using single-cell RNA-seq. *Nature* 509, 371-375. 10.1038/nature13173 24739965 PMC4145853

[DMM021626C49] van den Hout L., Schaible T., Cohen-Overbeek T. E., Hop W., Siemer J., van de Ven K., Wessel L., Tibboel D. and Reiss I. (2011). Actual outcome in infants with congenital diaphragmatic hernia: the role of a standardized postnatal treatment protocol. *Fetal Diagn. Ther.* 29, 55-63. 10.1159/000322694 21325859

[DMM021626C50] Vuckovic A., Roubliova X. I., Votino C., Naeije R. and Jani J. C. (2012). Signaling molecules in the fetal rabbit model for congenital diaphragmatic hernia. *Pediatr. Pulmonol.* 47, 1088-1096. 10.1002/ppul.22512 22328320

[DMM021626C51] Vuckovic A., Herber-Jonat S., Flemmer A. W., Roubliova X. I. and Jani J. C. (2013). Alveolarization genes modulated by fetal tracheal occlusion in the rabbit model for congenital diaphragmatic hernia: a randomized study. *PLoS ONE* 8, e69210 10.1371/journal.pone.0069210 23840910 PMC3698086

[DMM021626C52] Wang Y., Maciejewski B. S., Weissmann G., Silbert O., Han H. and Sanchez-Esteban J. (2006). DNA microarray reveals novel genes induced by mechanical forces in fetal lung type II epithelial cells. *Pediatr. Res.* 60, 118-124. 10.1203/01.pdr.0000227479.73003.b5 16864689

[DMM021626C53] Wu J., Yamamoto H., Gratacos E., Ge X., Verbeken E., Sueishi K., Hashimoto S., Vanamo K., Lerut T. and Deprest J. (2000). Lung development following diaphragmatic hernia in the fetal rabbit. *Hum. Reprod.* 15, 2483-2488. 10.1093/humrep/15.12.2483 11098015

[DMM021626C505] Xu B., Chen C., Chen H., Zheng S. G., Bringas P. Jr, Xu M., Zhou X., Chen D., Umans L., Zwijsen A. et al. (2011). Smad1 and its target gene Wif1 coordinate BMP and Wnt signaling activities to regulate fetal lung development. *Development* 138, 925-935. 10.1242/dev.062687 21270055 PMC3035095

[DMM021626C506] Yu X., Ng C. P., Habacher H. and Roy S. (2008). Foxj1 transcription factors are master regulators of the motile ciliogenic program. *Nat. Genet.* 40, 1445-1453. 10.1038/ng.263 19011630

